# Metabolomic analysis of gingival crevicular fluid for dental age and skeletal maturity assessment in growing individuals

**DOI:** 10.1093/ejo/cjag064

**Published:** 2026-09-18

**Authors:** Tsagkari Eirini, Panteris Eleftherios, Papadopoulos A Moschos, Chatzigianni Athina

**Affiliations:** Department of Orthodontics, Faculty of Dentistry, School of Health Sciences, Aristotle University of Thessaloniki, 54124 Thessaloniki, Greece; Biomic_AUTh, CIRI-AUTH Center for Interdisciplinary Research and Innovation, Aristotle University of Thessaloniki, 54124 Thessaloniki, Greece; Department of Orthodontics, Faculty of Dentistry, School of Health Sciences, Aristotle University of Thessaloniki, 54124 Thessaloniki, Greece; Department of Orthodontics, Faculty of Dentistry, School of Health Sciences, Aristotle University of Thessaloniki, 54124 Thessaloniki, Greece

**Keywords:** metabolomics, biomarkers, skeletal growth, orthodontics, dentistry, tooth development

## Abstract

**Objectives:**

The aim of this study was to examine the metabolic profile of growing patients for potential identification of biomarkers related to dental skeletal development.

**Materials and methods:**

Gingival crevicular fluid samples from 54 young patients were collected and further analyzed by an untargeted gas chromatography–mass spectrometry method. For the estimation of dental development, the Demirjian method was used, and for the skeletal maturation level, the cervical vertebrae maturation method was applied. Multivariate and univariate analyses were performed to examine possible metabolomic variations according to the chronological, dental, and skeletal age. Group differences were evaluated using Kruskal–Wallis tests, with Bonferroni correction applied to account for multiple comparisons. Spearman’s ρ correlation coefficient was used to examine variable correlations. Metabolite expression variations were visualized using boxplots.

**Results:**

In total, 63 compounds were retrieved with metabolomic analysis. The results showed that cadaverine and L-valine uniquely displayed robust dental age-related variation, demonstrating significant elevations during tooth development and decrease in the late stages of the dentition. Malonic acid and nonanoic acid decrease with advanced dental age. Regarding bone growth, cadaverine, L-serine, and L-homoserine have been identified as strong indicators of skeletal maturation.

**Conclusions:**

Several distinct metabolites showed coherent associations with skeletal and dental maturation. Additional studies employing complementary analytical methods and broader metabolome profiling are necessary to validate their association with tooth and bone growth pathways.

**Clinical significance:**

The adjunct use of metabolomics in dentistry and orthodontics may lead to more precise diagnosis and personalized healthcare.

## Introduction

In orthodontics, the diagnosis is mainly based on patient’s clinical and radiographic examination, while the evaluation of patient’s maturation level is important for treatment planning and treatment stability. Chronological age in orthodontics has been considered a nonreliable index for young patients’ maturation estimation, since individual variations in skeletal and dental development are usually evident. Several radiographic indices and methods have been proposed and applied to determine dental age and bone maturity. However, the overall biological basis of somatic and craniofacial growth and matureness is yet to be discovered.

Lately, in the medical field, a shift to basic research has led to the use of biological fluids with the purpose of identifying the biological profile of individuals offering a personalized diagnosis and treatment with the use of omics technology [1]. Likewise, the use of oral biofluids, such as saliva and gingival crevicular fluid (GCF), can lead to the era of precision dentistry and orthodontics.

In 1965, GCF was first used as an indicator of periodontal disease in humans [2], and further studies complemented the use of GCF in dental diagnostics [3, 4]. With regards to saliva, it appears that the first food and drug administration-cleared saliva collection devices, which enabled clinical validation of salivary diagnostics, date back to the 1990s [5].

Since then, several studies have proven the association between biomarkers and various clinical conditions or therapeutic interventions. In dentistry, periodontically compromised dentitions are the most frequently examined from a biological perspective. In a recent study, chronic periodontitis patients were presented with elevated levels of GCF or serum protein resistin as compared with healthy individuals. Resistin modulates inflammation in chronic periodontal disease and may be used as surrogate measure to identify subjects at risk for periodontitis [6]. A systematic review aimed to find evidence about the metabolic profile of the GCF in patients with periodontitis, and indeed there were small metabolites that were distinct among gingivitis, chronic periodontitis, and aggressive periodontitis. In addition, untargeted metabolomic analyses identified over 40 metabolites, primarily related to amino acid and lipid degradation pathways, that were significantly discriminant between periodontal conditions [7].

It is evident that, likewise in dentistry, the analysis of oral biofluids can be beneficial during every step of orthodontic procedure, from orthodontic diagnosis to the development of treatment plan, the monitoring of the response to treatment, and finally during the retention period. Targeted identification of specific biomolecules has been broadly applied until now in basic sciences to reveal biochemical secretions under certain conditions. Metabolome comprises a broad range of endogenous and exogenous compounds and can be affected by both genetic and environmental factors. It is a valuable source of biomolecules linked to biochemical pathways [8, 9]. In a paradigm shift in dentistry and orthodontics, the untargeted analysis and identification of the metabolome could reveal new diagnostic and therapeutic potentials.

For example, during the early stages of an orthodontic procedure, it is important to assess the optimal treatment timing and the efficacy of the proposed treatment plan. It is well known that in certain skeletal discrepancies, the child’s skeletal age plays an important role in selecting treatment alternatives. In this direction, there is evidence that targeted analysis of salivary alkaline phosphatase (ALP) activity, which has already been conducted in several studies, is a promising diagnostic tool for prepubertal growth phase prediction [10, 11]. Targeted investigations of protein insulin-like growth factor 1 have also been applied to find possible association with the pubertal growth spurt [12, 13]. Nevertheless, the identification of specific proteins in oral biofluids and the available evidence for accurate pubertal growth prediction are limited and sometimes contradictory. Possible new insights could be offered with the untargeted metabolomic analysis of the oral biofluids.

Thus, the aim of this study was to conduct an untargeted metabolomic analysis of the GCF in growing patients in order to correlate specific metabolites with individual’s dental development and pubertal growth phase.

## Materials and methods

### Sample

The study sample initially comprised 54 young patients (34 females and 20 males) seeking orthodontic treatment at the University Postgraduate Orthodontic Clinic. Three participants were excluded from the final analytical sample because the collected GCF volume was insufficient for metabolomic analysis. Therefore, the final analytical sample comprised 51 participants (32 females and 19 males) (Supplementary Table S1). The research protocol received approval from the Ethics Committee of the corresponding Faculty of Dentistry. Written informed consent was obtained from the patients’ guardians prior to enrollment and before the initiation of orthodontic treatment.

Eligibility criteria for participation included: (i) age between 8 and 16 years, (ii) mixed or permanent dentition with complete eruption of the upper central and lateral incisors, (iii) overall good health with no systemic diseases, (iv) no medication use for at least 1 month before entering the study, (v) good periodontal condition, with plaque and bleeding indices below 25%, and (vi) no history of previous or ongoing orthodontic treatment.

All participants underwent a periodontal examination and received professional ultrasonic scaling 2 weeks prior to GCF collection. In addition, they were instructed to maintain proper oral hygiene and to refrain from eating or brushing their teeth for at least 90 min before GCF sampling.

### Collection procedures of gingival crevicular fluid

Firstly, GCF samples were collected with the use of PerioPaper® GCF Collection Strips (Oraflow Inc., NY, USA) inserted for 30 s in the periodontal pocket of healthy gingiva, which were then directly transferred to an Eppendorf tube and stored at −80°C until further analysis.

### Chronological age subgroups

The patients’ sample was categorized according to the chronological age of the participants. Patients up to 10 years old were considered to belong to Group 1 (children), patients 11–12 years old formed Group 2 (early adolescence), and patients 13–16 years old formed Group 3 (late adolescence).

### Dental age assessment and subgroups

Dental age was estimated by means of the method of Demirjian, using panoramic radiographs. According to this method, three subgroups were formed, namely Group 1, with dental age up to 10 years old; Group 2, with dental age 11–12 years old; and Group 3, with dental age 13–16 years old (Supplementary Table S1).

### Skeletal age assessment and subgroups

Panoramic and lateral cephalometric radiographs were routinely taken prior to the initiation of orthodontic treatment, with a maximum interval of 1 week from the collection of GCF. Skeletal maturity was assessed using the cervical vertebral maturation (CVM) method on the lateral cephalograms, following the guidelines established by Baccetti *et al.* [14]. The evaluation was independently performed by two clinicians, with one conducting a second assessment after a 2-week interval. Based on the CVM stages, patients were classified into three groups reflecting different phases of skeletal development. The method outlines six stages (CS1–CS6) for growth prediction. Patients at stages CS1 and CS2 were assigned to Group 1 (prepubertal group), those at stages CS3 and CS4 to Group 2 (pubertal group), and individuals at stages CS5 and CS6 to Group 3 (postpubertal group) (Supplementary Table S1).

### Sample analysis

Low molecular weight metabolites were extracted by soaking the Periopaper® in 450 μL of a solvent mixture (MeOH:H_2_O:CHCl_3_ = 2.5:1:1). After ultrasonic extraction for 5 min, the solution was centrifuged at 16 000*×g* for 5 min at 4°C. The supernatants were dried under vacuum and subjected to a two-step derivatization: first with methoxyamine hydrochloride (MeOx) in pyridine, followed by N-Methyl-N-(trimethylsilyl)trifluoroacetamide containing 1% Trimethylchlorosilane. The derivatized extracts were analyzed in an EVOQ 456 GC-TQ-MS triple quadrupole system (Bruker) equipped with a CTC-PAL autosampler (CTC Analytics AG) on a 30 m Agilent J&W HP-5ms column using a 42-min analytical run. The details of the analytical protocol applied are provided in our previous work [15].

### Data analysis

Chromatographic data were processed using MSWS software (Bruker Daltonics) and converted into Net.CDF (.CDF) format. Compound identification was carried out using the automated mass spectral deconvolution and identification system (AMDIS), which referenced two spectral libraries: the Agilent Fiehn Metabolomics Spectral Library (Fiehn) and the National Institute of Standards and Technology Spectral Library. AMDIS analysis was conducted in simple mode, with a minimum match threshold set at 50%. Deconvolution parameters included medium resolution and sensitivity, moderate shape criteria, and a component width of 20 mass spectral scans. The output consisted of (.FIN) and (.ELU) files containing the extracted library spectra.

These (.CDF), (.ELU), and (.FIN) files were then further processed using the assignment validator and integrator (GaVin) script for MATLAB (MathWorks, Natick, MA, USA). Visual inspection for both compound identification and peak integration was conducted using the Fiehn library in combination with the GaVin script. All patient subgroups—based on skeletal, dental, and chronological age—were included in the analysis.

To assess group differences, both univariate and multivariate statistical analyses were applied to the normalized data matrix, incorporating the internal standard. Statistical significance was determined using a two-tailed Student’s *t*-test assuming equal variances (homoscedastic) in Excel365, with a significance level set at *P* < 0.05. Fold changes in metabolite levels were expressed as base-2 logarithmic ratios (log_2_FC). Variations in metabolite expression were visualized using boxplots. For multivariate analysis, principal component analysis was conducted using SIMCA 13.0 (Umetrics).

Statistical analysis was conducted using IBM SPSS Statistics for Windows, version 26 (IBM Corp., Armonk, NY, USA), and Microsoft Excel. Clinical, procedural, and demographic variables are presented as mean ± standard deviation or as frequencies and percentages, as applicable. Given the nonparametric nature of the data, group differences were assessed using Kruskal–Wallis tests with Bonferroni correction applied for multiple comparisons. Spearman’s ρ correlation coefficients were computed to explore associations between variables. Additional statistical analyses, including generation of boxplots and heat maps, were performed using Python (version 3.11) with pandas (version 1.3.3), scipy.stats (version 1.7.1), and matplotlib (version 3.4.3) libraries. Statistical significance was set at *P* < 0.05.

## Results

Untargeted gas chromatography-mass spectrometry (GC–MS) screening initially yielded a broad pool of derivatized features (63 putative metabolites). To enhance biological interpretability, we applied *a priori* curation criteria: removal of clear derivatization artifacts and reagent by-products, consolidation of duplicate derivatives, and retention of compounds with plausible endogenous or oral microbiome relevance. After filtering, 33 metabolites were carried forward as the analytic panel (Supplementary Table S2 and Fig. S1). All inferential testing and figures in the main text are based on this biologically grounded set.

### Differences according to gender

**Figure 1:**
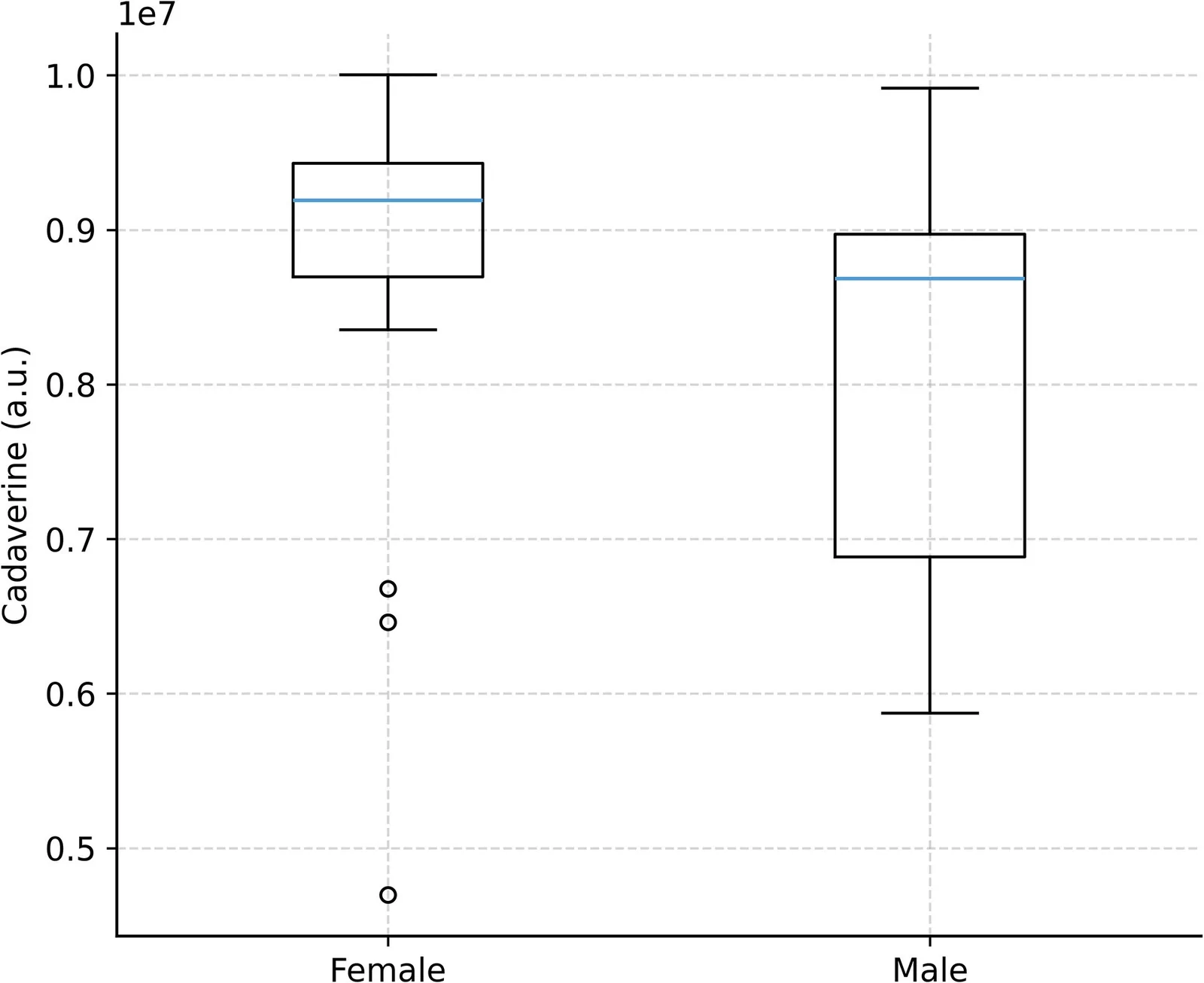
Cadaverine abundances across sex. Boxes span the interquartile range; center lines mark medians; whiskers extend to 1.5× IQR; circles denote outliers. *Y*-axes show raw ion counts (a.u.).

Among all analytes, only cadaverine differed by sex. Cadaverine concentrations were higher in females than males (Mann–Whitney *U* test, *P* = 0.013). Group medians with interquartile ranges (IQRs) are tabulated in Table 1; the corresponding single-panel boxplot is shown in Fig. 1.

**Table 1:** Sex comparison (significant metabolites).

Metabolite	1 median (IQR)	2 median (IQR)	Mann–Whitney *P*	Higher group
**Cadaverine**	9 191 282 (8 695 935–9 431 483)	8 683 735 (6 884 441–8 972 797)	0.013	Female

Values are raw abundances.

### Chronological age

No analyte reached the α = 0.05 threshold across chronological age groups. Borderline patterns (0.05 < *P* < 0.10) were noted for L-valine, L-homoserine, cadaverine, glycine, and aspartic acid; summary medians and *P* values are provided in Table 2. These trends are exploratory and warrant confirmation in larger samples. All medians (IQRs), exact *P* values and Bonferroni-adjusted pairwise comparisons, are reported in Table 2.

**Table 2:** Chronological age subgroups (borderline metabolites, 0.05 < *P* < 0.10).

Metabolite	Group 1 median (IQR)	Group 2 median (IQR)	Group 3 median (IQR)	Kruskal–Wallis *P*
**Aspartic acid**	56 848 (26 960–109 590)	37 087 (24 603–75 740)	22 463 (15 739–33 922)	0.078
**Cadaverine**	9 193 986 (8 606 538–9 269 003)	8 806 988 (7 099 938–9 088 360)	7 936 546 (6 788 928–8 774 275)	0.074
**Glycine**	2 716 797 (1 635 590–4 657 228)	1 993 734 (1 092 520–3 552 101)	1 053 864 (913 741–2 428 639)	0.089
**L-Homoserine**	7 440 734 (6 998 851–7 906 933)	6 820 989 (6 465 576–7 301 120)	7 121 252 (6 600 242–7 872 098)	0.069
**L-Valine**	309 358 (258 163–484 025)	234 761 (143 330–421 814)	129 848 (114 127–238 417)	0.056

Values are raw abundances; medians (IQR). Kruskal–Wallis *P* from three-group comparison; none survived α = 0.05.

### Dental maturation

Dental age group comparisons (Group 1, Group 2, and Group 3) identified multiple metabolites exhibiting significant stage-related differences post-Bonferroni adjustment. Malonic acid (propanedioic acid) was highest in Group 1 relative to Groups 2 and 3 (overall *P* = 0.001; Group 1 > Group 2 and Group 1 > Group 3 after Bonferroni adjustment). Cadaverine and L-valine were higher in Group 2 than in groups 1 and 3 (both with significant Bonferroni-adjusted pairwise contrasts). Nonanoic acid showed a significant difference between Group 1 and Group 2. Phosphoric acid, benzoic acid, and oleic acid met the overall Kruskal–Wallis threshold but did not yield surviving pairwise contrasts after correction.

**Figure 2:**
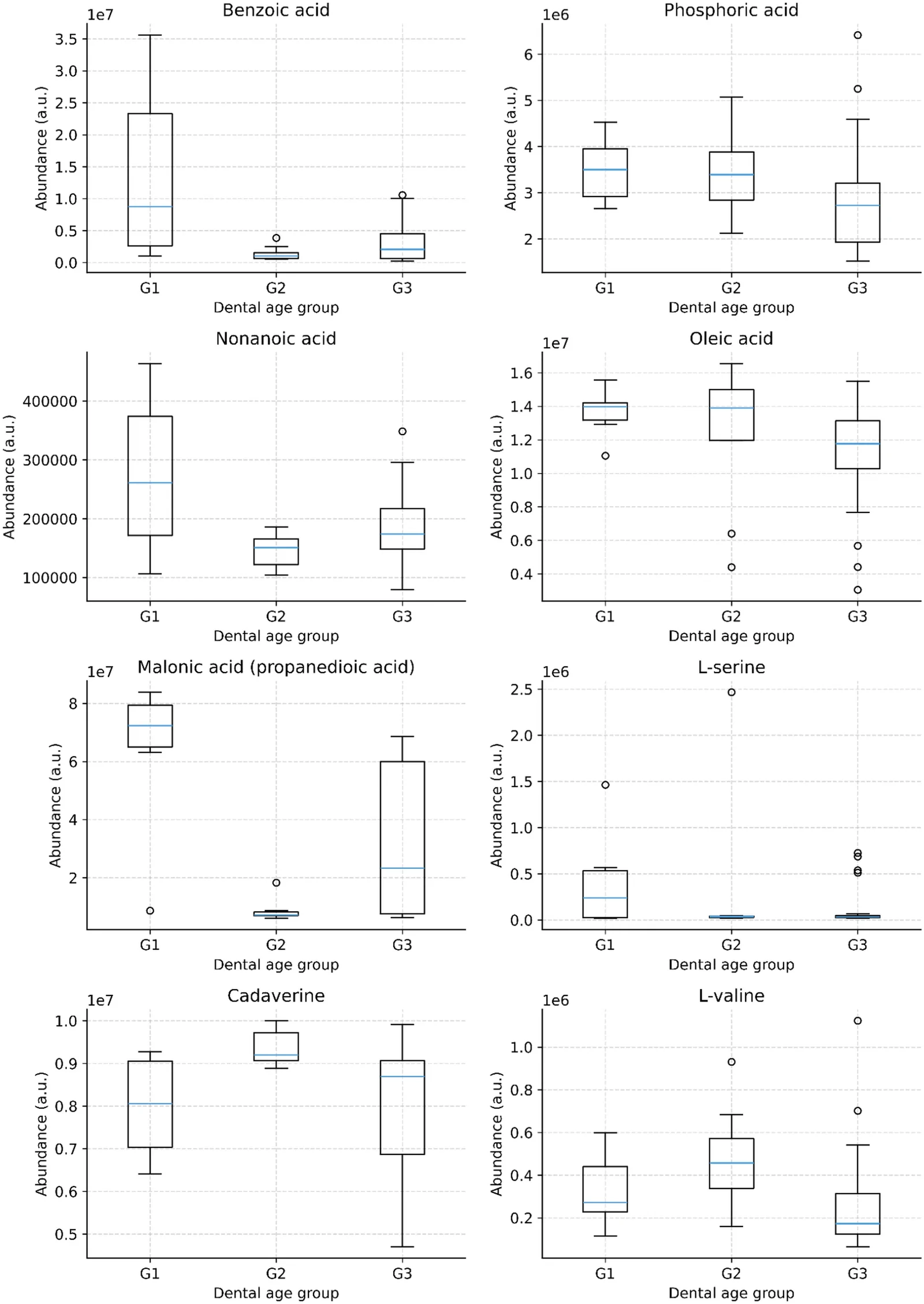
Metabolite abundances across dental-age groups (≤10, 11–12, 13–16 years). Boxes span the interquartile range; center lines mark medians; whiskers extend to 1.5× IQR; circles denote outliers. *Y*-axes show raw ion counts (a.u.).

Exact medians, IQRs, and adjusted pairwise *P* values are presented in Table 3; eight corresponding boxplots (panels A–H) are displayed in Fig. 2.

**Table 3:** Dental age subgroups—Kruskal–Wallis with Bonferroni pairwise (significant metabolites only).

Metabolite	Group 1 median (IQR)	Group 2 median (IQR)	Group 3 median (IQR)	Kruskal–Wallis *P*	Pairwise *P* (Bonferroni)
**Benzoic acid**	8 745 823 (2 636 093–23 287 026)	1 034 171 (643 199–1 557 228)	2 053 770 (628 468–4 527 450)	0.048	G1-G2: *P* = 0.036
**Phosphoric acid**	3 500 389 (2 917 990–3 953 485)	3 391 084 (2 839 919–3 883 324)	2 724 207 (1 926 165–3 207 628)	0.048	
**Nonanoic acid**	261 152 (171 534–373 953)	150 830 (122 128–165 736)	174 028 (148 615–217 135)	0.035	G2-G3: *P* = 0.040
**Oleic acid**	13 978 271 (13 169 009–14 218 119)	13 900 199 (11 955 101–14 988 192)	11 775 362 (10 289 293–13 134 548)	0.032	NS
**Malonic acid (propanedioic acid)**	72 347 043 (64 990 047–79 374 819)	7 185 484 (7 034 965–8 179 421)	23 238 660 (7 538 824–59 998 982)	0.001	G1-G2: *P* = 0.005 G1-G3: *P* = 0.005
**L-Serine**	24 688 (22 046–33 607)	42 930 (35 185–62 581)	34 135 (28 585–40 154)	0.030	G2-G3: *P* = 0.030
**Cadaverine**	8 057 708 (7 031 936–9 055 092)	9 196 689 (9 064 772–9 726 695)	8 690 874 (6 865 771–9 066 505)	0.009	G2-G3: *P* = 0.006
**L-Valine**	272 168 (228 707–439 862)	457 170 (338 201–571 508)	173 268 (124 608–314 254)	0.011	G2-G3: *P* = 0.013

Pairwise *P* values are Mann–Whitney *U* with Bonferroni adjustment; only adjusted *P* < 0.05 are reported.

### Skeletal maturation

**Figure 3:**
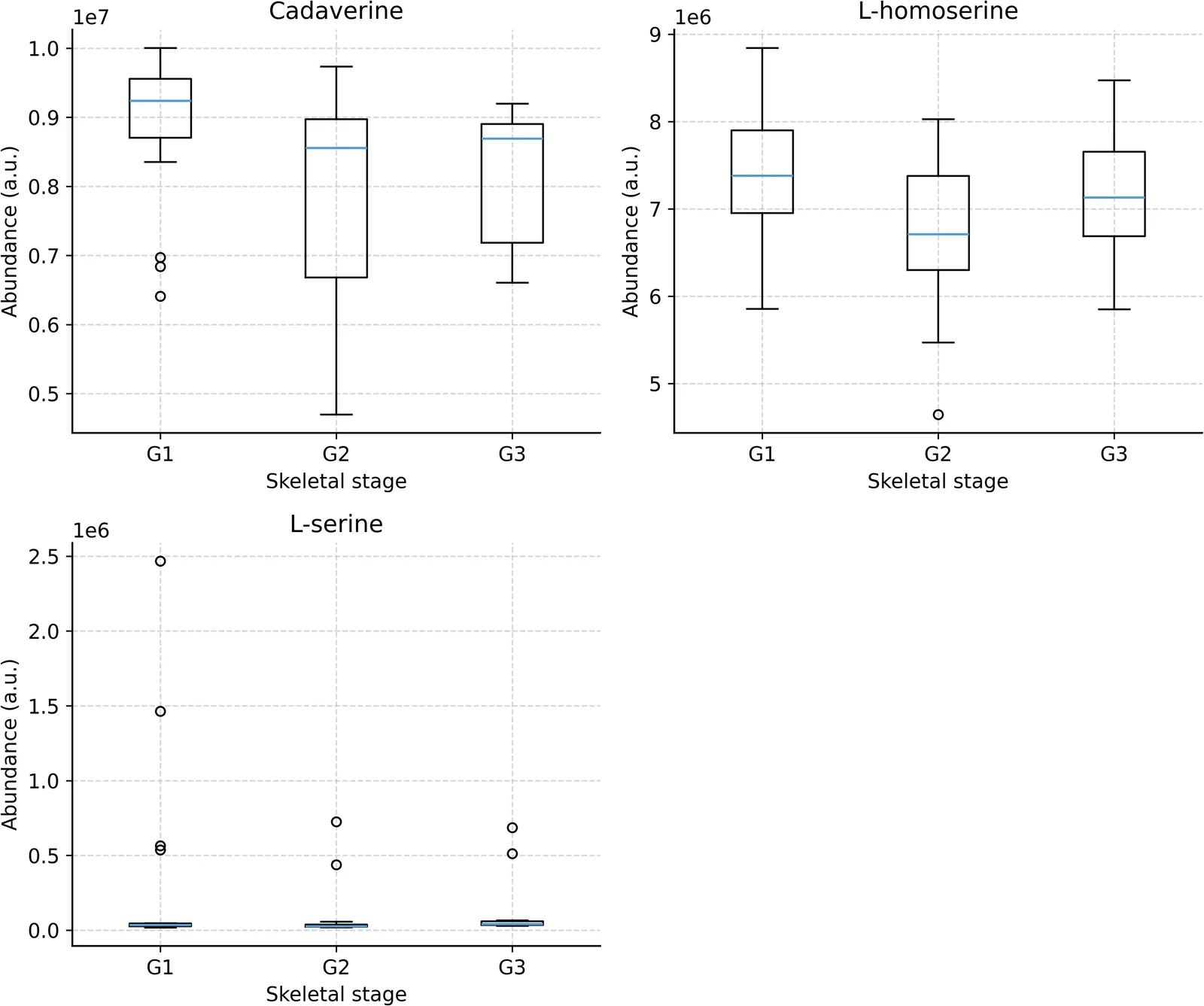
Metabolite abundances across skeletal-age groups (CS1–2, CS3–4, CS5–6). Boxes span the interquartile range; center lines mark medians; whiskers extend to 1.5× IQR; circles denote outliers. *Y*-axes show raw ion counts (a.u.).

Metabolite distribution across skeletal age groups revealed significant differences for three metabolites following Bonferroni correction. Cadaverine decreased from the earliest to later stages (Kruskal–Wallis *P* = 0.007, with Bonferroni-adjusted pairwise differences; Table 4). L-Homoserine also showed stage-related variation (overall *P* = 0.033), and L-serine differed across groups (overall *P* = 0.034). Medians (IQRs) and significant pairwise contrasts are reported in Table 3; boxplots for significant metabolites appear in Fig. 3.

**Table 4:** Skeletal age subgroups—Kruskal–Wallis (significant metabolites only).

Metabolite	Group 1 median (IQR)	Group 2 median (IQR)	Group 3 median (IQR)	Kruskal–Wallis *P*	Significant pairwise (Bonferroni)
**Cadaverine**	9 236 389 (8 704 609–9 556 867)	8 555 777 (6 681 928–8 973 693)	8 690 874 (7 183 806–8 903 692)	0.007	G1-G2 (*P* = 0.016); G1-G3 (*P* = 0.042)
**L-Homoserine**	7 378 900 (6 952 482–7 898 823)	6 708 590 (6 298 268–7 377 983)	7 129 728 (6 686 657–7 654 752)	0.033	G1-G2 (*P* = 0.033)
**L-Serine**	34 345 (25 705–45 366)	26 005 (22 485–37 343)	40 327 (35 201–60 299)	0.034	G3-G2 (*P* = 0.020)

Values are raw abundances. Pairwise tests use Mann–Whitney *U* with Bonferroni correction; only adjusted *P* < 0.05 are reported.

NS, nonsignificant.

### Correlations

**Figure 4:**
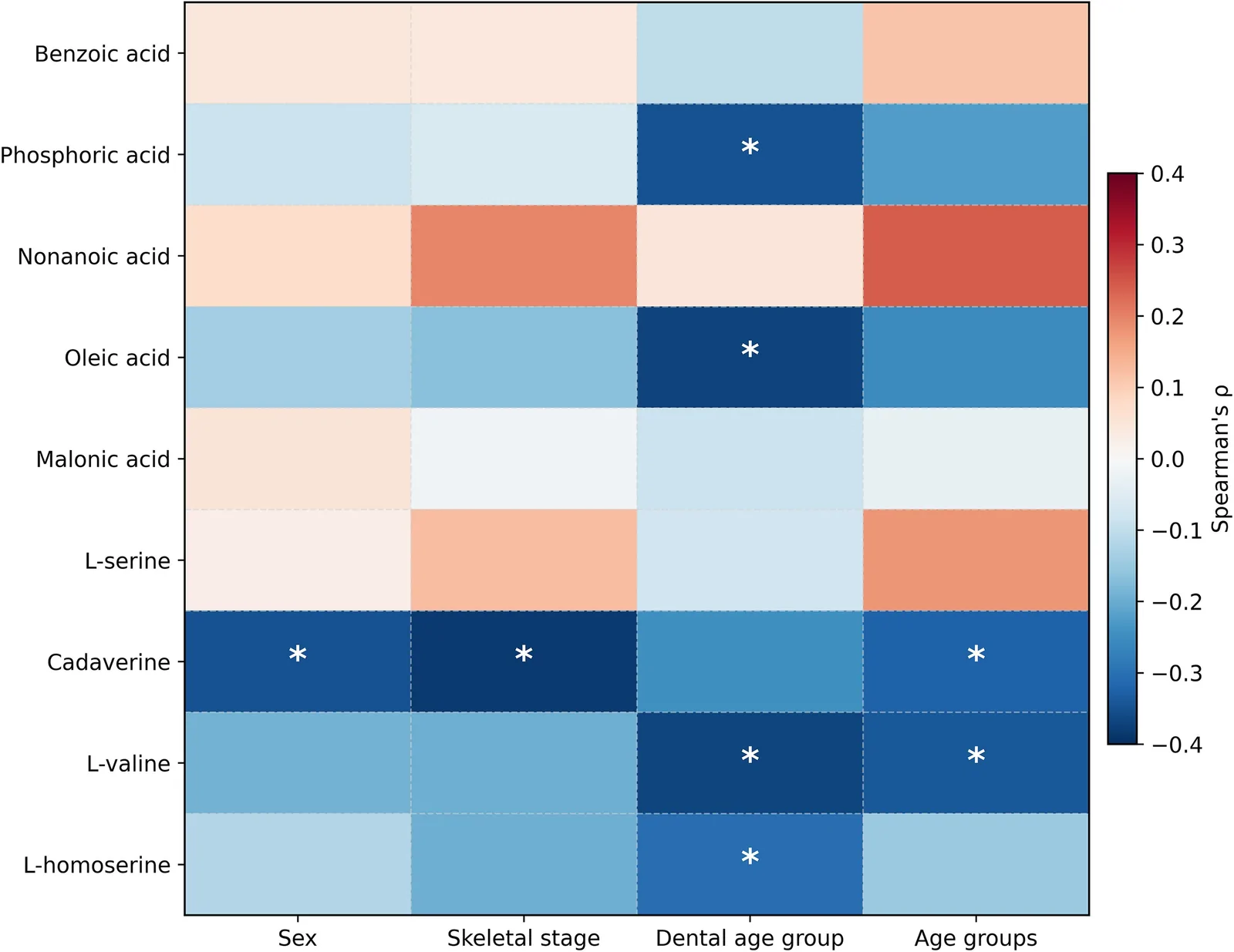
Heat map of metabolite correlations (*P < 0.05).

The correlation heat map (Fig. 4) indicates a coherent pattern of modest inverse associations between maturational status and several metabolites. Cadaverine declines with advancing skeletal stage (*ρ* = −0.383, *P* = 0.006) and chronological age (*ρ* = −0.323, *P* = 0.021) and showed a significant association with sex, with higher concentrations observed in males than in females. Among amino acids, L-valine correlates inversely with both dental group (*ρ* = −0.367, *P* = 0.008) and chronological age (*ρ* = −0.340, *P* = 0.015), while L-homoserine is negatively associated with dental group (*ρ* = −0.304, *P* = 0.030). Oleic acid and phosphoric acid also decrease with later dental maturation (*ρ* ≈ −0.35; ≈0.01). No comparably strong positive correlations were detected. Overall, the heat map patterns accord with the nonparametric group comparisons: indices related to polyamine turnover and amino-acid metabolism tend to diminish as dental and skeletal development progresses, whereas other metabolites (e.g. malonic acid) show group differences without a strictly monotonic trend across ordered stages (Fig. 4).

## Discussion

In the present study, differentiated distribution of several metabolites among chronological, dental, and skeletal age subgroups was evident, while correlations between metabolite levels and chronological, dental, and skeletal age subgroups, as well as sex, were proved.

To date, no GCF metabolomic studies have been performed to investigate the dental or skeletal maturation in children. The study focused on a biologically curated panel and discussed only the metabolites that remained significant in the final analyses and figures: cadaverine, L-valine, L-serine, L-homoserine, and malonic acid (propanedioic acid), with additional stage-related signals for benzoic acid, phosphoric acid, nonanoic acid, and oleic acid. This restrained scope follows the principle that untargeted outputs should be interpreted through a biologically plausible lens and cross-checked against orthogonal evidence [1, 8, 9].

Cadaverine was the only sex-dependent metabolite observed and, beyond sex effects, showed lower levels with advancing skeletal stage. This pattern is consistent with the view that polyamine flux mirrors tissue remodeling intensity, higher earlier and then tapering as growth slows. Nevertheless, bone formation (osteogenesis) is known to be regulated by osteoblast-lineage programs (e.g. Wnt/β-catenin, bone morphogenetic protein/RUNX2) and not by cadaverine. Even though cadaverine has mostly bacterial origin, humans and other animals can produce small amounts of cadaverine enzymatically. Trace levels can be part of normal metabolic processes and polyamine pathways. *In vitro*, other polyamines, such as putrescine, spermidine, and spermine, can promote osteogenic differentiation of human mesenchymal stem cells s and are involved in cell growth, differentiation, and DNA stabilization [16]. Potentially, cadaverine may act as a metabolic by-product of amino acid catabolism and play minor roles similar to other polyamines. In some cases, cadaverine is useful as a biomarker candidate (saliva/urine) in inflammatory conditions (e.g. periodontal disease; exploratory in inflammatory bowel disease)) [17].

In GCF, such signals likely track local matrix dynamics and cell turnover rather than calendar time *per se*, aligning with long-standing evidence that GCF reflects periodontal and peri-tooth biology with high temporal sensitivity [3, 4, 18].

Across skeletal staging, L-serine and L-homoserine varied with maturation, and L-valine differentiated in later dental groups and correlated inversely with ordered dental/age indices. Together, these amino-acid changes are compatible with shifts in anabolic demand and intermediary metabolism during the pubertal window, which can be captured in oral fluids [10, 19]. L-Serine is involved in endochondral ossification during long-bone growth [20], while serine pathway is required for osteoclast and osteoblast differentiation [21, 22].

Regarding L-valine, untargeted metabolomics of human dental follicles show age-related pathway shifts (steroid/bile acid, carbohydrate, and lipoate metabolism), underscoring a metabolic component to follicle biology [23]. In the broader oral/periodontal stem-cell context, branched-chain amino acids can modulate cell behavior showing these amino acids are bioactive in oral mesenchyme [24].

Although the present data are preliminary, the directionality—lower metabolite levels with more advanced stages—fits the broader literature showing that biomarker profiles evolve as growth decelerates and remodeling becomes less intense [7].

Malonic acid was highest in the youngest dental group and lower thereafter, suggesting early-stage engagement of dicarboxylic-acid-linked intermediary pathways. Cell-permeable malonic acid inhibits succinate dehydrogenase, reshaping macrophage metabolism, and inflammatory signaling [25], while succinate signaling intersects bone/periodontal biology [26].

Among lipid-related features, nonanoic acid distinguished early from mid dental groups, whereas oleic acid showed an overall effect without surviving pairwise contrasts; oleic acid in general promotes osteogenic differentiation (RUNX2, osteocalcin, ALP, mineralization) in periodontal ligament fibroblasts, suggesting a pro-formation tilt in dental mesenchyme similar to dental follicle-derived osteoblasts [27]. Phosphoric acid and benzoic acid likewise reached overall significance with modest, nonmonotonic trends.

The above overall findings, given the known influence of diet and microbiome on many low-molecular-weight polyamines, aminoacids, and lipids detectable by GC-MS, should be treated as hypothesis-generating and verified with targeted assays [1, 7].

The heat map corroborated the group tests, showing predominantly negative correlations between maturation indices and polyamine/amino-acid abundances, with no comparably strong positive trends. Notably, some metabolites (e.g. malonic acid) exhibited clear group differences without strictly monotonic rank-order correlations, implying that stage transitions rather than linear trajectories may drive part of the signal—an observation that fits how biological maturational staging behaves in clinical orthodontics [19].

### Limitations and future directions

This study is an initial, cross-sectional GC–MS investigation with derivatization chemistry and a modest sample size. Also, *a priori* sample size calculation was not feasible due to the novelty of the method. Moreover, dietary habits and oral microbiome composition were not assessed. The final analytical sample was modest, and some subgroup sizes were relatively small and imbalanced, particularly in the dental age classification; therefore, the present findings should be considered exploratory and hypothesis-generating rather than definitive evidence of validated biomarkers. Additionally, the use of CVM method for skeletal age assessment may induce issues compared to hand-wrist radiographic evaluation. Several signals are therefore provisional. Multiple-comparison control reduces false positives but also limits power for pairwise contrasts, and some effects (benzoic, phosphoric, oleic acids) remain at the “overall-only” level. The immediate priority is targeted quantification of the short list (cadaverine, L-valine, L-serine, L-homoserine, malonic acid) in larger, preferably longitudinal cohorts, ideally integrating microbial and dietary covariates. Such work would test reproducibility and clarify whether these metabolites can augment skeletal/dental staging during orthodontic diagnosis and monitoring.

## Conclusion

In this preliminary, biologically curated GC–MS analysis of GCF, a small set of metabolites-cadaverine and the amino acids, L-valine, L-serine, and L-homoserine-showed coherent associations with skeletal and dental maturation, while malonic acid, and to a lesser extent benzoic, phosphoric, nonanoic, and oleic acids, displayed stage-related differences. The overall pattern was a downward shift in polyamine and amino-acid signals with advancing maturation, consistent across group comparisons and correlation structure, with a single sex effect for cadaverine. These findings support the feasibility of GCF metabolomics as a complementary readout of craniofacial growth, but they remain exploratory and warrant caution. Priorities for translation include quantification in larger, longitudinal cohorts, integration of dietary and microbiome covariates, and assessment of incremental value over existing dental and skeletal staging. If validated, biomarker panels could augment orthodontic diagnosis, timing, and monitoring with objective, minimally invasive measures.

## Supplementary Material

cjag064_Supplementary_Data

## Data Availability

The datasets generated and analyzed in the current study are available from the corresponding author upon reasonable request.
