# Supplementary material for: Metabolomic analysis of gingival crevicular fluid for dental age and skeletal maturity assessment in growing individuals
Source: Eur J Orthod. 2026 Sep 18;48(5):cjag064. doi: 10.1093/ejo/cjag064 (PMC13587827; doi:10.1093/ejo/cjag064)
Supplement: Supplementary file 1 [file cjag064_supplementary_data.zip › Supplementary file.docx]

**Supplementary tables**

**Table S1:** Distribution of the final analytical sample of 51 participants by chronological, dental and skeletal age subgroups.

| **Category** | **Group** | **Total (n)** | **Male (n)** | **Female (n)** |  |
| --- | --- | --- | --- | --- | --- |
| Chronological Age | 1 | 14 | 8 | 6 | |
|  | 2 | 26 | 10 | 16 | |
|  | 3 | 11 | 1 | 10 | |
| Dental Age | 1 | 6 | 3 | 3 | |
|  | 2 | 9 | 7 | 2 | |
|  | 3 | 36 | 9 | 27 | |
| Skeletal Maturation | 1 | 19 | 12 | 7 | |
|  | 2 | 20 | 7 | 13 | |
|  | 3 | 12 | 0 | 12 | |

**Table S2:** Retention time (RT) and retention index (RI) data for the analyzed 33 compounds. The calculated RI values are compared with reference values obtained from the NIST database, and the percentage difference (% Diff) between calculated and literature RI values is provided to assess identification accuracy.

|  | **Compound** | **RT** | **RI calculated** | **RI NIST** | **% Diff** |
| --- | --- | --- | --- | --- | --- |
| 1 | L-Lactic acid | 7.22 | 1104 | 1061 | 4.06 |
| 2 | Glycolic acid | 7.40 | 1107 | 1072 | 3.26 |
| 3 | Ethanolamine | 8.05 | 1118 | 1021 | 9.51 |
| 4 | Oxalic acid | 8.42 | 1125 | 1116 | 0.79 |
| 5 | L-Alanine | 8.96 | 1135 | 1110 | 2.27 |
| 6 | 2-Hydroxybutyric acid | 9.36 | 1143 | 1130 | 1.16 |
| 7 | p-Cresol | 9.42 | 1144 | 1153 | -0.75 |
| 8 | **L-homoserine** | 9.44 | 1145 | 1197 | -4.35 |
| 9 | Pentanoic acid, 2-(trimethylsilyloxy)-trimethylsilyl ester | 10.10 | 1159 | 1207 | -3.99 |
| 10 | **L-valine** | 11.46 | 1191 | 1229 | -3.12 |
| 11 | Pyruvic acid | 12.05 | 1206 | 1249 | -3.47 |
| 12 | **Benzoic acid** | 12.35 | 1214 | 1229 | -1.26 |
| 13 | Caprylic acid (octanoic acid) | 12.90 | 1229 | 1266 | -2.96 |
| 14 | Leucine/Isoleucine | 13.00 | 1231 | 1285 | -4.18 |
| 15 | **Phosphoric acid** | 13.08 | 1234 | 1286 | -4.08 |
| 16 | L-Proline | 13.40 | 1243 | 1302 | -4.56 |
| 17 | **Glycine** | 13.54 | 1247 | 1317 | -5.34 |
| 18 | Glyceric acid | 14.65 | 1280 | 1344 | -4.74 |
| 19 | **L-serine** | 14.96 | 1290 | 1368 | -5.69 |
| 20 | **Nonanoic acid** | 15.07 | 1294 | 1355 | -4.52 |
| 21 | **Malonic acid** | 16.70 | 1349 | 1425 | -5.34 |
| 22 | **Cadaverine** | 17.68 | 1385 | 1391 | -0.45 |
| 23 | **Aspartic acid** | 19.35 | 1450 | 1536 | -5.57 |
| 24 | Lactamide MEA | 19.36 | 1451 | 1323 | 9.67 |
| 25 | 5-Oxoproline | 22.02 | 1567 | 1522 | 2.99 |
| 26 | Dodecanoic acid | 23.75 | 1651 | 1556 | 6.10 |
| 27 | Tetradecanoic acic | 26.17 | 1779 | 1752 | 1.51 |
| 28 | Hexadecanoic acid | 30.09 | 2011 | 1954 | 2.89 |
| 29 | Myo-Inositol | 31.03 | 2071 | 2152 | -3.77 |
| 30 | Octadecadienoic acid (Z,Z) | 32.51 | 2170 | 2212 | -1.91 |
| 31 | **Oleic acid** | 32.60 | 2176 | 2208 | -1.47 |
| 32 | Octadecanoic acid | 32.90 | 2196 | 2239 | -1.90 |
| 33 | Uridine | 34.82 | 2333 | 2469 | -5.51 |

*RT: retention time; RI: retention index; NIST: database; Bold: annotated; NIST RI depending on TMS derivative*

**Supplementary figure legends**

**Figure S1:** Relationship between experimental retention time (RT) and theoretical retention index (RI) used for compound identification. The calibration curve was generated using standard compounds, with the fitted polynomial regression model and coefficient of determination (R²) indicated. The inset shows the RT–RI relationship for the standards used to construct the calibration. This model was applied to calculate RI values for detected compounds and assess their agreement with reference values.
